# Protein kinase CK2α is overexpressed in classical hodgkin lymphoma, regulates key signaling pathways, PD-L1 and may represent a new target for therapy

**DOI:** 10.3389/fimmu.2024.1393485

**Published:** 2024-05-14

**Authors:** Edoardo Ruggeri, Federica Frezzato, Nayla Mouawad, Marco Pizzi, Federico Scarmozzino, Guido Capasso, Valentina Trimarco, Laura Quotti Tubi, Alessandro Cellini, Chiara Adele Cavarretta, Valeria Ruocco, Andrea Serafin, Francesco Angotzi, Nicolò Danesin, Sabrina Manni, Monica Facco, Francesco Piazza, Livio Trentin, Andrea Visentin

**Affiliations:** ^1^ Hematology Unit, Department of Medicine (DIMED), University of Padova, Padova, Italy; ^2^ Surgical Pathology and Cytopathology Unit, Department of Medicine, University of Padova, Padova, Italy

**Keywords:** classical hodgkin lymphoma, anti-CD30, MMAE, CK2, PD-L1

## Abstract

**Introduction:**

In classical Hodgkin lymphoma (cHL), the survival of neoplastic cells is mediated by the activation of NF-κB, JAK/STAT and PI3K/Akt signaling pathways. CK2 is a highly conserved serine/threonine kinase, consisting of two catalytic (α) and two regulatory (β) subunits, which is involved in several cellular processes and both subunits were found overexpressed in solid tumors and hematologic malignancies.

**Methods and results:**

Biochemical analyses and *in vitro* assays showed an impaired expression of CK2 subunits in cHL, with CK2α being overexpressed and a decreased expression of CK2β compared to normal B lymphocytes. Mechanistically, CK2β was found to be ubiquitinated in all HL cell lines and consequently degraded by the proteasome pathway. Furthermore, at basal condition STAT3, NF-kB and AKT are phosphorylated in CK2-related targets, resulting in constitutive pathways activation. The inhibition of CK2 with CX-4945/silmitasertib triggered the de-phosphorylation of NF-κB-S529, STAT3-S727, AKT-S129 and -S473, leading to cHL cell lines apoptosis. Moreover, CX-4945/silmitasertib was able to decrease the expression of the immuno-checkpoint CD274/PD-L1 but not of CD30, and to synergize with monomethyl auristatin E (MMAE), the microtubule inhibitor of brentuximab vedotin.

**Conclusions:**

Our data point out a pivotal role of CK2 in the survival and the activation of key signaling pathways in cHL. The skewed expression between CK2α and CK2β has never been reported in other lymphomas and might be specific for cHL. The effects of CK2 inhibition on PD-L1 expression and the synergistic combination of CX-4945/silmitasertib with MMAE pinpoints CK2 as a high-impact target for the development of new therapies for cHL

## Introduction

Classical Hodgkin’s lymphoma (cHL) is an uncommon malignancy of the lymphatic system, usually affecting young adults (1, 2). Although the treatment of patients with cHL has improved with the combination or the sequential use of drugs targeting CD30 [such as the anti-CD30 monoclonal antibody conjugated with the monomethyl auristatin E (MMAE) brentuximab vedotin] and immune checkpoint PD-1 inhibitors (such as pembrolizumab and nivolumab) with chemotherapy, the outcome of double or triple refractory patients is still poor. Therefore, new targeted therapies with innovative mechanisms of action and new drug combinations are urgently needed (3–5). The mononuclear Hodgkin’s cells alongside the prominent presence of large, bi- or multinucleated Reed-Sternberg (HRS) cells, which account for less than 2% of the total tumor bulk, display a constitutive pattern of activated signaling pathways due to EBV infection, microenvironment interaction and gene mutations that drive to activation of among others, NF-κB (nuclear factor kappa B), JAK/STAT3 (Janus Kinase/Signal transducer and activator of transcription factor 3), and PI3K/Akt (Phosphoinositide 3-kinase/RACα serine/threonine protein kinase) pathways (6). For some of these pathways clinically inhibitors have already been developed and used in different hematological malignancies [i.g., PI3K/Akt inhibitors (7) or JAK1/2 inhibitor (8–10)]. Additionally, these oncogenic pathways regulate the expression of PD-L1 (programmed death ligand 1). Through its interaction with PD-1, PD-L1 assumes a pivotal role in modulating mechanisms related to immunosuppression and T-cell exhaustion (5).

Protein CK2 is a constitutively active and highly conserved serine/threonine kinase, that has reached increasing visibility as a potential pharmacological target (5, 11). Structurally, CK2 is a tetrameric holoenzyme, composed of two catalytic α and/or α′, and two regulatory β subunits, in the possible configurations α2β2, αα′β2, or α′2β2 (12). The CK2 α and β dimers may also be present as dimer which seems to associated with distinct and specific functions (13). It is well documented that CK2 is involved in a wide range of biological processes, such as cell proliferation, differentiation, apoptosis and DNA damage repair (14, 15). Our group has demonstrated that both CK2α and CK2β subunits are overexpressed in acute leukemias, multiple myeloma and non-Hodgkin lymphomas (15–18). CK2 mediates the phosphorylation of NF-κB p65 (RelA) directly on Serine 529 (S529), of STAT3 on S727 and of AKT on S129, promoting the survival of neoplastic cells as well as drug resistance (19).

In this study we investigated the expression of CK2α and CK2β in a panel of HL cell lines and HL patient samples, analyzed CK2 mediated activation of survival signaling pathways and investigated the capability of inhibiting CK2 with the ATP-competitive inhibitor CX-4945/Silmitasertib along with MMAE to trigger HRS cell apoptosis and assess PD-L1 expression levels. These findings present a novel potential target to overcome resistance or to increase MMAE cytotoxicity.

## Methods

### Cell cultures and treatments

L-428, L-540, HDLM-2, and KM-H2 HL cell lines were kindly provided from Prof. Carmelo Carlo-Stella from Humanitas Hospital (Milan, Italy). The expression of the immuno-phenotypic markers of the HL lines is reported in the **Supplementary Table S1**. Testing for Mycoplasma infection was carried at a monthly basis. As positive controls, we used Kasumi-1 cells, an M2 acute myeloid leukemia cell line (Liebniz Institute Germane Collection of Microorganism and Cell Cultures DSMZ, Germania). Healthy donor B cells, serving as the normal control, were isolated from buffy-coat using EasySep™ kits (STEMCELL Technologies, USA). Two×10^6^ cells for each HL cell line were resuspended in 1ml of appropriate culture medium and plated in 12/24-well plates. Cells were incubated at different time points (24, 48, and 72 hours) with medium only or with CX-4945/Silmitasertib 0, 5, 10, and 15μM (provided from Selleck Chemicals, Munich - Germany) or with MMAE 5nM (20) (monomethyl auristatin E; Selleck Chemicals). For specific experiments HL cell lines were also treated (2x10^6^/ml) with the proteasome inhibitor Bortezomib (BTZ) 10nM up to 36 hours (Selleck Chemicals).

### Flow cytometry

Aliquots of 100×10^5^ HL cell lines were harvested, washed in PBS 1x, and incubated for 10 min in the dark at room temperature and co-stained with the following antibodies: CD20 (APC-H7 conjugated), CD30 (PE conjugated) (Becton Dickinson; Franklin Lakes, NJ, USA), CD274/PD-L1 (PE-Cy7 conjugated, Fisher Scientific; Hampton, NH, USA). After incubation with antibody, cells were washed with PBS and analyzed by the flow cytometry. For each sample, 20,000 events were acquired and analyzed using the FACSCanto II™ A cytometer and data were processed using the DIVA Software (Becton Dickinson). For each antibody the mean fluorescence intensity was reported as compared to the untreated condition.

### Evaluation of apoptosis

Apoptosis of different cell samples (pathological cells from different HL cell lines, normal B lymphocytes) were assessed using the Annexin V/Propidium Iodide (PI) staining (Apoptosis Detection Kit, Valter Occhiena, Turin, Italy), and by detection of PARP cleavage in western blotting (21).

### Assessment of drug concentration-effect and calculation of the combination index

L-428, L-540, HDLM-2, and KM-H2 HL cell lines were plated into 48 well plates (2×10^5^ cells/ml) in appropriate culture medium. CX-4945, MMAE were added at different concentrations: CX-4945 ranging from 1μM to 25μM and of MMAE ranging from 0.05nM to 10nM for 72h alone or in combination. Cell viability was measured through Trypan blue exclusion dye assay. The concentration of the single drug able to kill the 50% of cells (EC50) was determined by fitting the dose-response curve utilizing the Prism 7 software (GraphPad Software Inc. La Jolla, CA, USA). To calculate the Combination Index, cells were treated with a combination of CX-4945 and MMAE using the method of constant ratio drug combination proposed and described by Chou and Talalay (22).

### Western blot and antibodies

Whole cell extracts (WCE) were obtained by RIPA Lysis and extraction buffer (Tris-HCL 20mmol/l, NaCl 150mmol/l, EDTA 5.0mmol/l, Niaproof 1.5%, Na3VO4 1.0mmol/l, SDS 0.1%, Thermo Fisher Scientific, Waltham, MA, USA), added with protease inhibitors (Halt Protease Inhibitor Cocktail, Thermo Fisher Scientific), phosphatase inhibitors (Phosphatase Inhibitor Cocktail, Thermo Fisher Scientific), and EDTA (Thermo Fisher Scientific) on ice. Nuclear and cytoplasmic Subcellular Fractionation were prepared using a commercial kit (Thermo Scientific, Rockford, IL, USA). The supernatant was quantified by BCA protein quantification assay (Thermo Fisher Scientific). Equal amounts of protein sample were added to 3× Red loading sample buffer (Cell Signaling Technology, Danvers, USA) and boiled for 5 min. Western blotting was conducted according to standard protocols (23). Briefly, 15 to 25 μg of WCE or nuclear and cytoplasmic fractions were subjected to SDS-PAGE, transferred to Nitrocellulose/PVDF membranes and immunoblotted with the following primary antibodies: anti-Akt-Ser129or anti-Akt-Ser473 (Cell Signaling), anti-Akt, anti-STAT3-Ser727, anti-STAT3 (Abcam, Cambridge, UK), anti-CK2β and anti-CK2α (kindly provided by Prof. Maria Ruzzene, University of Padova), anti-NF-κB-Ser529 (p65) (Cell Signaling), anti-NF-κB-p65 (Abcam), anti-PARP (Cell Signaling), mono- and polyubiquitinylated protein conjugates (Enzo Life Science Ltd, Exeter, UK), anti-β-actin (Sigma-Aldrich, St. Louis, MO), anti-α-tubulin (Sigma Aldrich), anti-GAPDH (Cell Signaling). Detection was performed using chemiluminescence reaction (ECL, Euroclone, Milan, Italy). Images were acquired using the Amersham Imager 600 (GE Healthcare; Chicago, IL, USA), the protein bands were scanned and quantified by densitometry, using the Image J program (Github, San Francisco, USA).

### Immunoprecipitation

HL cell lines (L-540, L-428, HDLM-2, KM-H2) were lysed in Blast R™ Lysis Buffer by Signal-Seeker™ Kit (Cytoskeleton-Thermofisher Scientific) and the enriched total ubiquitinated proteins from HL cell line lysates were pulled out by an affinity beads system. Ubiquitinated protein fractions were obtained from 1mg total protein lysates. After extensive washes in IP buffer the immunocomplex was resuspended in Laemmli buffer with β-mercaptoethanol and processed for WB analysis.

### Confocal microscopy analysis

Aliquots of the different cell samples (pathological cells from different HL cell lines and normal B lymphocytes from healthy subjects) were collected, washed and plated on poly-L-lysine coated slides for 15 min at RT. Cells were then fixed in 4% paraformaldehyde for 10min, washed twice with PBS 1x and permeabilized with 0,1% Triton X-100 (Sigma-Aldrich) for 4 min (24). Before staining, non-specific protein binding were blocked by incubating the slides for at least 30 min in 2% BSA. Cells were then stained with antibodies against CK2α and CK2β (the same used for WB) and DAPI for nuclear staining, washed three times with PBS and incubated with anti-mouse-Alexa488 secondary antibody for 30 minutes in the dark. Slides were mounted with cover slips and fluorescence was detected using the UltraView LCI confocal system (Perkin Elmer; Waltham, MA, USA) equipped with a fluorescence filter set for excitation at 488 and 360nm.

### Tissue microarray

Tissue microarrays were prepared from cases with adequate diagnostic material, as previously reported (25). In details, tumor areas enriched in HRS cells were selected and 3 tissue cores (diameter=1 mm) were obtained from each donor block. Appropriate positive and negative controls were also included. The tissue microarray was prepared by using the Galileo TMA CK3500 arrayer (Integrated System Engineering; Milan – Italy. Immunohistochemical staining for CK2α (EP1963Y, Epitomics, CA, USA) and CK2β (PA5-27416, ThermoFisher, Massachusetts, USA) were performed in duplicate. Antigen retrieval was performed with heat/ethylenediamine-tetra-acetic acid (EDTA) in an automated immunostainer (University Hospital of Padova). All cases with discordant immunohistochemical results were assessed in joint sessions at the microscope by two haemato-pathologists. The positivity for CK2α and CK2β were graded as: 0 = negative; 1 = positive <30% of HRS; 2 = positive 30-60% HRS or week-moderate intensity; 3 = positive >60% HRS or strong diffuse intensity. The clinical features, including age, sex and therapies of the 25 analyzed patients are summarized in **Supplementary Table S2**. Inclusion criteria were diagnosis of cHL before 2000 and having signed the informed consent. This part of the study was conducted according to the declaration of Helsinki, approved by the ethic committee of the Padova University Hospital (protocol # 4,089/AO/17) and informed consents were collected.

### Quantitative real-time PCR

Total cellular RNA was extracted from the four HL cell lines, Kasumi, and purified healthy B lymphocytes, using the RNeasy Mini Kit (Qiagen, Germany). cDNA was generated by Reverse Transcription System (Promega, USA). SYBR Green (FastStart Universal SYBR Green Master, ROX) real-time polymerase chain reaction (RT-PCR) was carried out in a Sequence Detection System 7000 (Applied Biosystem) with the ABI PRISM 7000 software (AppliedBiosystems, Foster City, CA). The primers used are the following: *CSNK2A1* (CK2α): Forward 5′-3′ GTTTGGGTTGTATGCTGGCA and Reverse 5′-3′ TTTCGAGAGTGTCTGCCCAA; *CSNK2A2* (CK2α’): Forward 5′-3′ CGACCATCAACAGAGACTGACTG and Reverse 5′-3′ GTGAGACCACTGGAAAGCACAG; *CSNK2B* (CK2β): Forward 5′-3′ TCCTTACCAACCGTGGCATC and Reverse 5′-3′ CATGCACTTGGGGCAGTAGA; *ACTIN* (β-actin): Forward 5′-3′ CCAGCTCACCATGGATGATG and Reverse 5′-3′ ATGCCGGAGCCGTTGTC.

### Statistical analysis

Statistical analyses were performed using Prism 7 (GraphPad Software Inc. La Jolla, CA, USA) for paired Student’s *t* test, *Kruskall-Wallis*, and *Mann-Whitney Test*, *Wilcoxon matched-pairs signed rank test*. Data are reported as mean ± standard deviation (SD). *p*<0.05 or less were considered statistically significant.

## Results

### CK2 subunits are unbalanced in cHL

**Figure 1:**
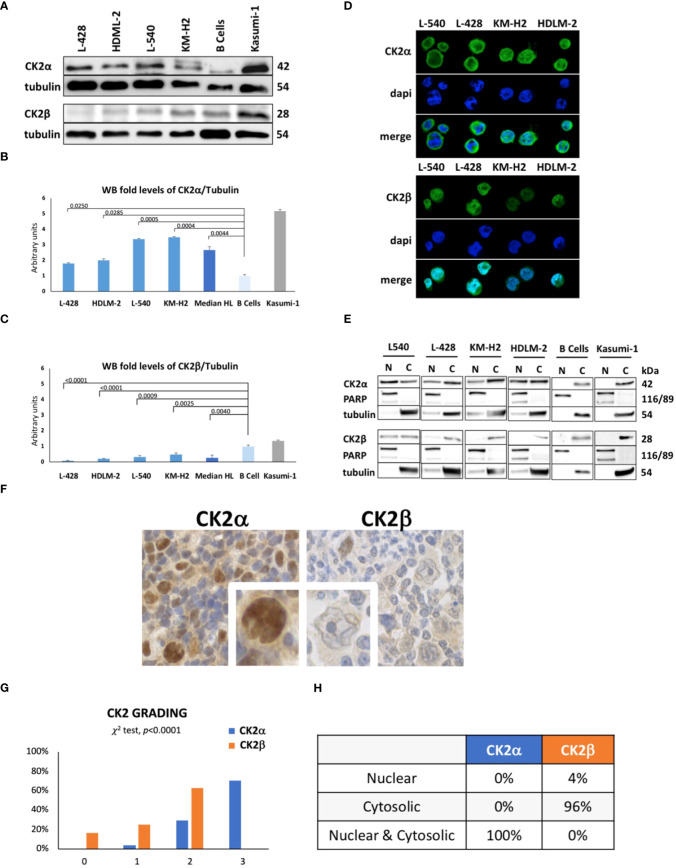
Protein expression levels of CK2 alpha (α) and beta (β) subunits in HL cell lines. Representative WB analysis **(A)**, and the corresponding densitometric values **(B, C)** are reported as mean ± SD of n= 3 independent experiments. α-Tubulin was used as the loading control. (L-428, HDLM-2, L-540, KM-H2 are HL cell lines; B cell are healthy lymphocytes purified from three different buffy coats; Kasumi-1, a cell line model of acute myeloid leukemia, was used ad positive control). Above the histogram bars **(B, C)**, the *p*-value is reported. In the upper-right part of the figure confocal microscopy is shown **(D)**. The experiment was performed in different preparations for each HL cell line; the α and β subunits of CK2 were detected using green fluorescence (Alexa Fluor 488), nuclei were visualized through blue staining (DAPI), and the merge for each CK2 subunit is shown. Images were collected with 40X magnification, Scale bars = 15μm. Cell Fractionation was performed on the four HL cell lines, healthy B cells (normal control), and Kasumi-1 cell line as positive controls. WB reports α and β subunits of CK2, PARP (Poly (ADP-ribose) polymerase) used as nuclear marker, α-Tubulin as cytosolic marker. The image reports a representative case of three independent experiments **(E)**. Expression and localization of CK2 α and β subunits assessed by tissue microarray performed in neoplastic lymph nodes from 25 patients and 5 reactive adenopathy **(F)**. Representative case of CK2 expression in patient-derived Hodgkin and Reed-Sternberg (HRS) cells. CK2α appears to be localized in both the nucleus and cytoplasm of HRS cells. Expression of CK2 subunits according to the grading level **(G)**, and description of their cellular localization **(H)**. Positivity was graded as: 0 = negative; 1 = positive <30% of HRS; 2 = positive 30-60% HRS or week-moderate intensity; 3 = positive >60% HRS or strong diffuse intensity.

We assessed expression of CK2 subunits in cHL cell lines. By WB, we found that all the four HL-derived cell lines L-428, L-540, KM-H2 and HDLM-2 expressed higher levels of the catalytic subunit CK2α than normal B lymphocytes (**Figure 1A**). As positive control, we stained protein extracts from Kasumi-1 cell line, which notably shows high expression levels of both the CK2 subunits (26). The median densitometry of CK2α/Tubulin for each cell lines was 0.46 ± 0.05 (*p*=0.0250), 0.51 ± 0.10 (*p*=0.0285), 0.86 ± 0.05 (*p*=0.0005), 0.89 ± 0.05 (*p*=0.0044) and 0.26 ± 0.09 for L-428, HDLM-2, L-540, KM-H2 and normal B-cell from heathy donors (**Figures 1A, B**). The median CK2α/Tubulin ratio in HL cell lines was 2.7-fold higher than healthy B lymphocytes (*p*=0.0044) (**Figure 1B**). Conversely, the non-catalytic subunit CK2β was found to be expressed at lower levels as compared to normal B lymphocytes (*p*<0.05) (**Figure 1A**). The median densitometry of CK2β/Tubulin was 0.07 ± 0.03 (*p*<0.0001), 0.20 ± 0.02 (*p*<0.0001), 0.31 ± 0.10 (*p*=0.0009), 0.47 ± 0.10 (*p*=0.0025) and 0.98 ± 0.09 for L-428, HDLM-2, L-540, KM-H2 and B-cell from heathy donors (**Figures 1A, C**). The median CK2β/Tubulin ratio in HL cell lines was 3.8-fold fewer than B lymphocytes (*p*=0.0040) (**Figure 1C**).

Immunofluorescence experiments (**Figure 1D**) revealed a strong and diffuse signal for CK2α in HL cells, while CK2β exhibited a lower intensity signal, particularly in the KM-H2 and HDLM-2 cell lines. CK2α seems to be localized both in the nucleus and cytosol of HRS cells, including the formation of discrete foci, as observed in the L-428 and KM-H2 cell lines. Instead, CK2β exhibits a diffuse and weak signal with a less distinct localization which hampers the quality of picture. In order to determine the subcellular distribution of α and β subunits more precisely in HL cell lines, we analyzed subcellular protein fractions, unveiling the presence of CK2α in both the nuclear and cytosolic compartments of HL cell lines. In contrast, CK2β predominantly localizes to the cytosol but exhibits a faint representation in the nuclear compartment of the L-540 and L-428 cell lines (**Figure 1E**).

To assess whether the dysregulation among CK2 subunits was linked to alterations in CK2 mRNA levels, we performed RT-PCR analysis on the mRNA of CK2 catalytic (α - *CSNK2A1* and α^I^ - *CSNK2A2*) and regulatory (β - *CSNK2B*) subunits. The mRNA levels in HL lines were then compared to those observed in control B lymphocytes. We observed statistically significant differences only for the CSNK2A1 gene, specifically in the L-428 (*p*<0.01) and L-540 (*p*<0.0001) cell lines (Supplementary Results, **Supplementary Figure S2C**).

### CK2 subunits are skewed in patients with HL

We performed a tissue microarray of neoplastic lymph nodes derived from 25 patients with cHL (**Supplementary Table S3**) and 5 with reactive adenopathies to evaluate the expression and localization of CK2 subunits in primary HL specimens. As shown in **Figure 1F, G** and **Supplementary Figures S1A–C**, we observed that 71% of patients strongly expressed CK2α in HRS cells (i.e., grade 3), while 29% were at grade 2 and 4% at grade 1 but no one was grade 0. Conversely, no patient expressed CK2β at grade 3, 67% of HL patients expressed CK2β at grade 2, 21% at grade 1 and 17% at grade zero (**Figures 1F, G**). Moreover CK2α was expressed both in the nucleus and the cytosol of all HRS, while CKβ was found in the cytosol in 96% of cases but in the nucleus only in 1 patient (*p*<0.0001) (**Figure 1H**, **Supplementary Figures S1A–C**) confirming our above-mentioned findings (**Figures 1D, E**). We also compared the clinical characteristics of our HL patients with the grading of CK2 subunits expression, but we did not find any correlation between age, gender, histological subtypes, interim 18-FDG PET-CT results, or relapsed cases. However, higher CK2β levels (i.e., grade 2) were associated with shorter progression free survival (PFS) than patient displaying lower levels (i.e., grade 0-1) (*p*=0.0421, **Supplementary Figure S2A**).

### CK2β subunit undergoes proteasome-dependent degradation

**Figure 2:**
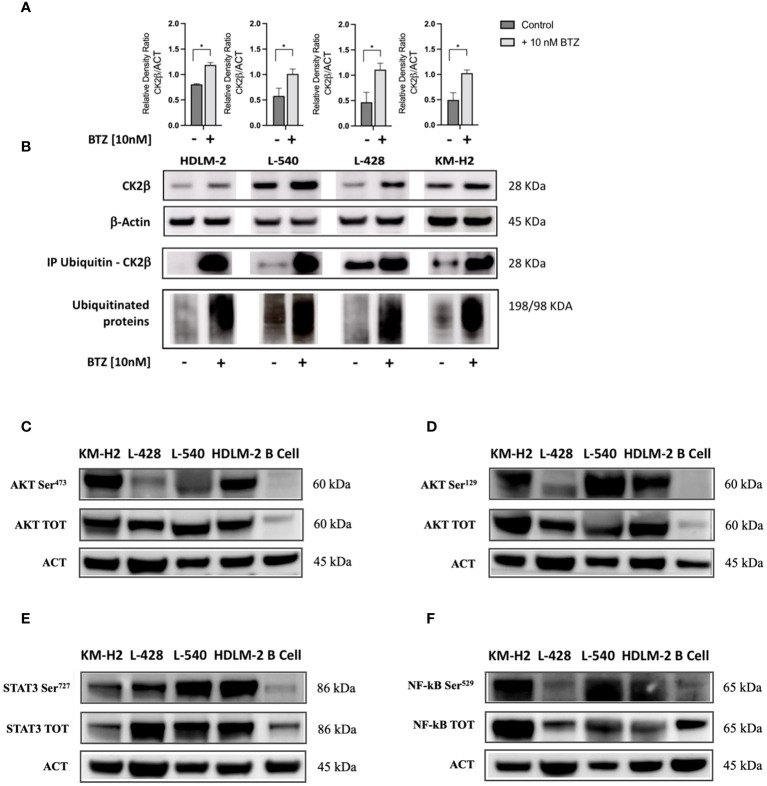
Western blotting analysis of ubiquitinated CK2β and phosphorylation levels of different CK2 targets. **(A)**. Relative densitometry of CK2β subunit with/without treatment of Bortezomib (BTZ) 10nM for 36h. **(B)** Representative WB. The proteins were immunoprecipitated, and all polyubiquitinated proteins were purified using a Ubiquitin detection kit. The immunocomplexes were then loaded onto an SDS-PAGE gel and subsequently analyzed using an anti-CK2β antibody. The results of proteasome inhibition were evaluated using the anti-poly ubiquitinated proteins (poly-Ubi) as positive control. For each condition, total cell lysates were loaded in SDS-PAGE and probed with anti-CK2β. * indicates *p*<0.05 (*Mann-Whitney test*), ACT: β-Actin; BTZ: Bortezomib. Representative WB of the main signaling pathways overexpressed in HL cell lines sustained by CK2 activity compared to the normal B lymphocytes **(C–F)**. The images depict phosphorylation levels at the Serine residue targeted by CK2α, and their corresponding total proteins for: AKT-S473 **(C)**, AKT-S129 **(D)**, STAT3-S727 **(E)**, and NF-kB (p65) S529 **(F)**. Densitometry for phosphorylated/total protein, total/β-Actin protein, and phosphorylated/β-Actin protein ratios are shown alongside each representative WB picture. β-Actin was used as the loading control. Analysis reports the mean ± SD of n = 3 independent experiments. * indicates *p*< 0.05 (*unpaired t test*).

To elucidate the mechanisms accounting for the downregulation of CK2β, we investigated whether CK2β was degraded by the proteasome. For this purpose, we treated HL cell lines *in vitro* with 10nM Bortezomib (BTZ) for 36 h. Inhibition of the proteasome resulted in an increase of the CK2β subunit protein levels compared to the untreated condition in the HL cell lines (**Figures 2A, B**). To further investigate this issue, all polyubiquitinated proteins from both the untreated and treated conditions were subjected to purification using specific affinity beads. The enriched protein samples were analyzed by WB, revealing a pronounced presence of polyubiquitinated CK2β in the BTZ-treated condition, strongly indicating proteasome-dependent degradation of β subunits in HL cell lines (**Figure 2B**). To verify if the proteasome was effectively inhibited, we assessed the nonspecific increase in polyubiquitin residues through WB, which demonstrated a marked increase of ubiquitinated proteins across a broad range of molecular weights, as expected. (**Figure 2B**).

### CK2 targets are phosphorylated

By means of WB analysis, we confirmed the overexpression of AKT, STAT3, and NF-kB (p65) as compared to their normal B cell counterparts (see **Figures 2C–F**). As depicted in the representative WB images, we found higher levels of both the whole protein and of the phosphorylated counterpart at the phosphorylation target sites of CK2α. Specifically, CK2 can phosphorylate AKT on Serine 473 (AKT S473, **Figure 2C**) by recruiting the mTOR2 complex (27) and directly phosphorylates AKT on Serine 129 (AKT S129, **Figure 2D**), NF-kB p65 on Serine 529 (NF-kB S529, **Figure 2E**), and STAT3 on Serine 727 (STAT3 S727, **Figure 2F**). These molecules exhibited phosphorylation at CK2-related residues under basal conditions, as compared to normal B lymphocytes. Protein phosphorylation levels diverged across different cell lines, reflecting the clinical heterogeneity observed in patients with HL. In addition, AKT, STAT3, and NF-kB (p65) were significantly more expressed in HL cell lines compared to normal B cells (*p*<0.05 unpaired Student’s *t* test, **Figures 2C–F**). Densitometric values have been summarized in **Supplementary Table S4**.

### CK2 modulates the expression of PD-L1 but not of CD30

**Figure 3:**
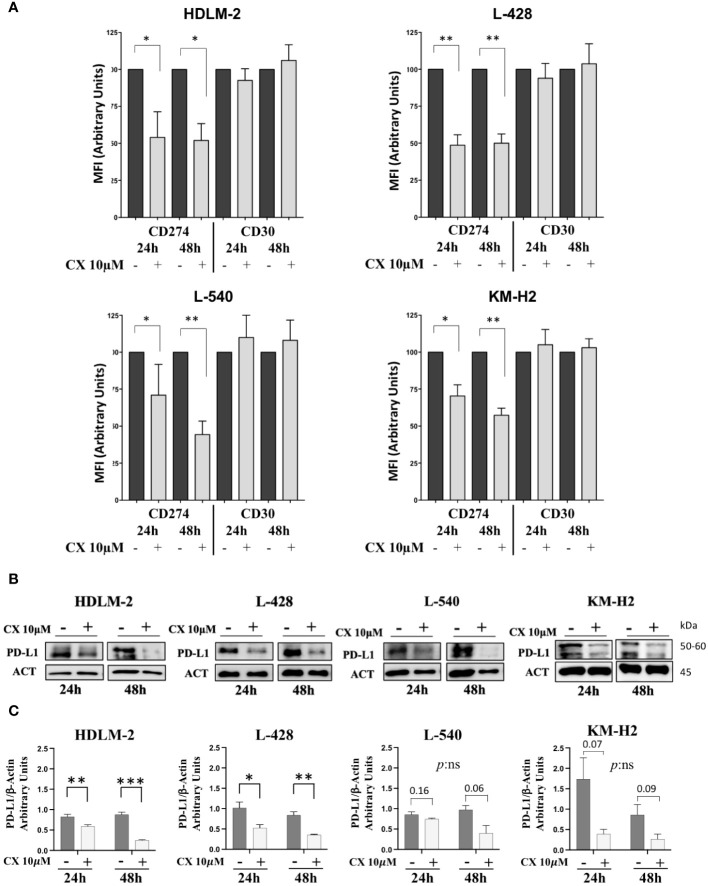
CD30 and CD274/PD-L1 expression assessment. In panel **(A)**, histograms depict the mean fluorescence intensity (MFI) of CD274 (PD-L1) and CD30 expression in the four HL cell lines, with or without 10μM CX-4945 treatment for 24 and 48 hours (three independent experiments, Student *t* test) CX: CX-4945. Only the viable cells were gated in the MFI analyses of PD-L1 or CD30 and normalized to their untreated condition. **(B)** Representative WB and **(C)** WB analysis of PD-L1 expression levels. HL cell lines were treated with or without 10μM CX-4945, for 24 and 48 hours. Densitometric values are reported as mean ± SD of n= 3 independent experiments, *Unpaired t test*. CX: CX-4945, ACT: β-Actin. *p<0.05, **p<0.01, ***p<0.001.

CD30 and PD-L1/CD274 are known to be expressed on the cell membrane of HRS cells, while CD20 is usually absent. According to data coming from the literature, in HRS the locus of the gene CD274, that is mapped on chromosome 9 is usually amplified (5). Moreover, STAT3, NF-kB, and AKT molecular pathways can regulate the expression PD-L1 gene (5, 28, 29). Considering the role of CK2 in activating all these proteins within cHL cell lines, we examined whether CK2 could potentially affect PD-L1 expression. To delve into this question, we treated HRS cell lines with CK2α-specific inhibitor CX-4945/silmitasertib at a concentration of 10μM for 24h and 48h and we assessed expression of CD20, CD30 and PD-L1. We found a decrease in the mean fluorescence intensity (MFI) of PD-L1 after treatment with CX-4945 in HL cell lines, while no significant changes were observed for CD20 and CD30 (**Figure 3A** and **Supplementary Figures S2B**). Specifically, after 24 hours of treatment, the decrease of CD274/PD-L1 MFI was 46.0% (p<0.05), 51.4% (p<0.01), 29.0% (p<0.05), and 29.6% (p<0.05), for HDLM-2, L-428, L-540, and KM-H2, respectively, as compared to untreated conditions. Similarly, after 48 hours of treatment, the CD274/PD-L1 MFI decreased of 31.3% (p<0.05) for HDLM-2, 50.0% (p<0.01) for L-428, 55.7% (p<0.01) for L-540, 42.6% (p<0.01) for KM-H2, compared to untreated conditions (Mann-Whitney test, **Figure 3A**). The downregulation of PD-L1 was also confirmed by WB analysis (**Figures 3B, C**). The PD-L1 expression values, determined through protein densitometry and normalized to β-actin, align with flow cytometry results, confirming a substantial reduction in protein levels following treatment with the CK2 inhibitor. Specifically, after 24 hours of culture, the PD-L1/β-actin ratios shifted from 1.17 **±** 0.14, 0.85 **±** 0.07, 1.74 **±** 0.5, and 0.82 **±** 0.06 under untreated conditions to 0.53 **±** 0.08, 0.74 **±** 0.02, 0.4 **±** 0.11, and 0.60 **±** 0.03 after treatment with CX-5945 at 10 μM, respectively, for L-428, L-540, KM-H2, and HDLM-2 cell lines. Specifically, HL cell lines L-428 and HDLM-2 displayed a significant difference in the means of the experimental triplicates, with p<0.05 and p<0.01, respectively (unpaired t test). The association between CK2 and PD-L1 became more evident after 48 hours of treatment. The PD-L1/β-actin ratios shifted from fold change values of 0.83 **±** 0.08, 1.0 **±** 0.1, 0.90 **±** 0.25, and 0.90 **±** 0.06 to values of 0.36 **±** 0.01, 0.4 **±** 0.18, 0.30 **±** 0.12, and 0.25 **±** 0.01, respectively, following treatment with CX-4945/silmitasertib at 10 μM for the L-428, L-540, KM-H2, and HDLM-2 cell lines. Once again, the L-428 and HDLM-2 cell lines exhibited a treatment-related difference that was statistically significant (p<0.01 and p<0.001, respectively, unpaired t test).

### CK2 inhibition triggers HL apoptosis

**Figure 4:**
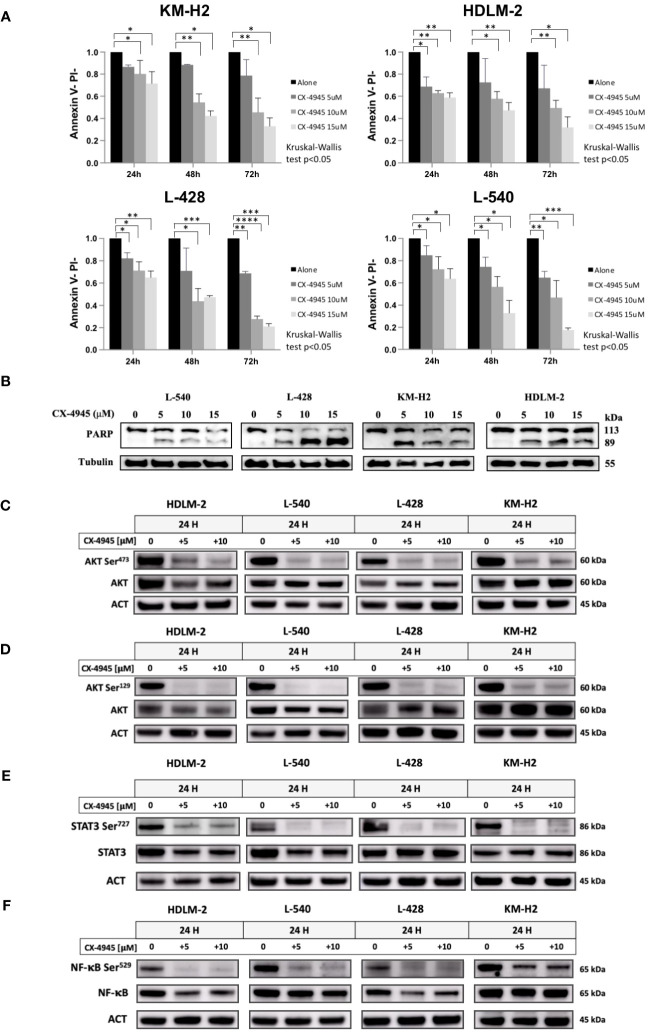
Apoptotic Effect of CX-4945 in HL Cell Lines. Apoptosis was detected through Annexin V propidium iodide (PI) assay by flow cytometry. Histograms illustrate the percentage of viable cells (Annexin V^neg^/PI^neg^) for each HL cell line after treatment with 5μM, 10μM, and 15μM CX-4945 at different time points (24, 48, and 72 hours). *p<0.05, **p<0.01, ***p<0.001, and ****p<0.0001 compared to the untreated cell population, *Kruskal-Wallis’s test* **(A)**. Qualitative WB was performed using anti-PARP to highlight apoptosis induction in all four HL cell lines. Protein lysates, obtained after 48 hours of treatment with 0, 5, 10, and 15μM CX-4945, are presented. α-Tubulin was used as the loading control (experimental duplicate) **(B)**. WB illustrate the impact of CK2 inhibition with CX-4945 on CK2 substrates in the four HL cell lines. WBs depict phosphorylation levels on CK2 Serine targets, with and without CX-4945 treatment, along with corresponding total protein levels. The panels illustrate phosphorylation levels on AKT S473 **(C)**, AKT S129 **(D)**, STAT3 S727 **(E)**, and NF-κB at S529 **(F)** at 0, 5, and 10μM CX-4945 after 24 hours of treatment (experimental duplicate). ACT: β-Actin.

Since CK2 is known to sustain pro-survival signals in cancer cells, we examined the effect of its chemical inhibition in HL cell line. Treatment with CX-4945/silmitasertib resulted in time- and dose-dependent apoptosis, as confirmed through Annexin V/Propidium Iodide flow cytometry testing (**Figures 4A, B**, *Kruskal-Wallis’s test*, *p*<0.05). For all the four HL cell lines, the percentage of viable cells exhibited a proportional decrease, starting from a dose of 5µM, in comparison to DMSO-treated cells (*p*<0.05). Notably, *in vitro* treatment with silmitasertib at 10µM concentration for 48 hours reduced the number of viable HL cells by half (**Figure 4A**). Furthermore, to elucidate the mechanisms underlying silmitasertib-induced apoptosis, we exposed HL cell lines to increasing doses of silmitasertib for 24 hours. This treatment resulted in the cleavage of PARP, a recognized marker of apoptosis (**Figure 4B**). Furthermore, that after 24 hours of cell treatment with CX-4945 (at concentrations of 5μM and 10μM), there was a significant decrease in the phosphorylation levels of AKT-S473 (**Figure 4C**), AKT-S129 (**Figure 4D**), STAT3-S727 (**Figure 4E**), and NF-kB-S529 (**Figure 4F**).

### CK2 inhibition boosts the activity of MMAE

Since CD30 levels were not affected after CK2 inhibition (**Supplementary Figure S2B**), we subsequently proceeded to investigate whether CX-4945/silmitasertib might enhance the effectiveness of this innovative therapy. Specifically, we investigated whether CK2 inhibition could enhance the cytotoxicity of MMAE, the microtubule inhibitor conjugated to the anti-CD30 monoclonal antibody brentuximab vedotin.

**Figure 5:**
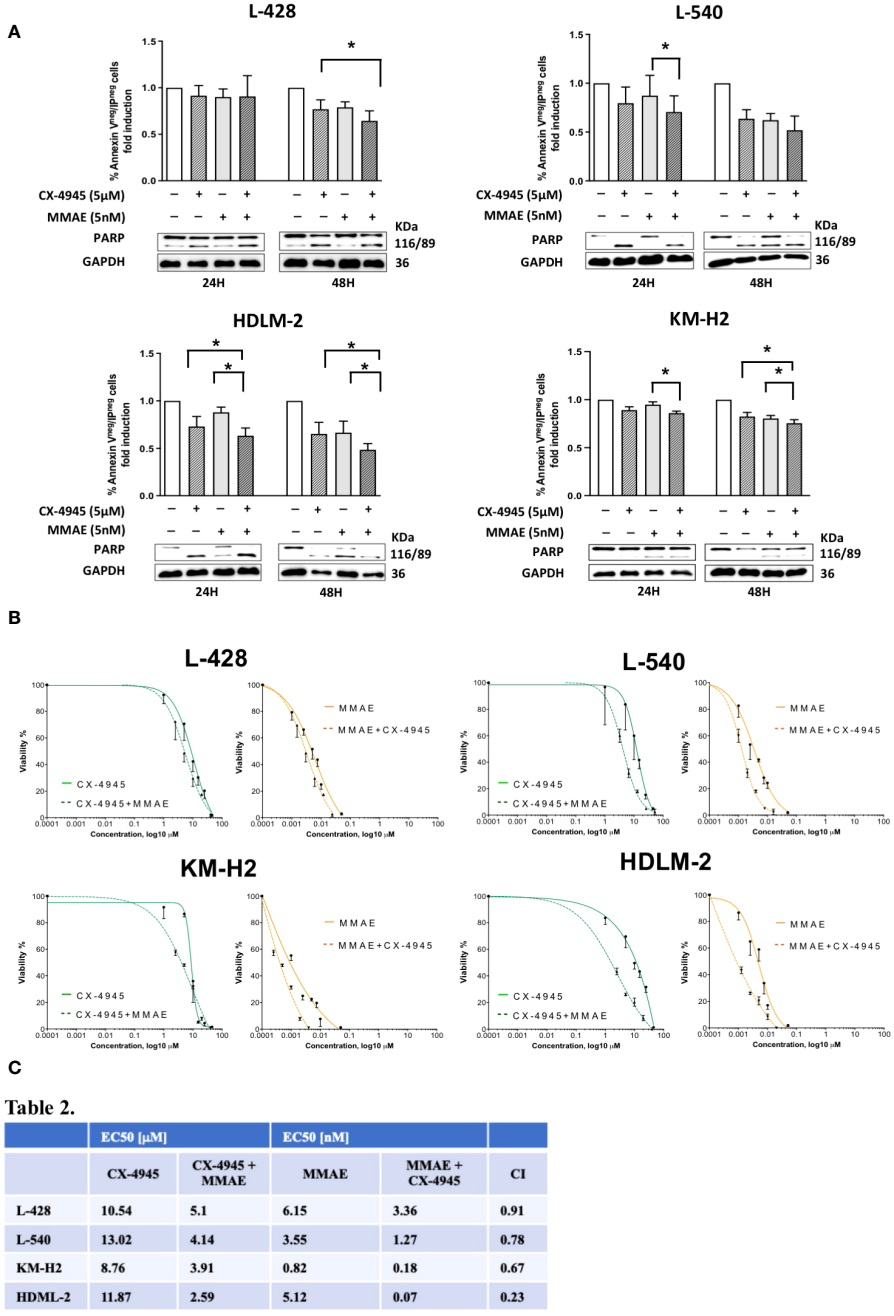
Apoptotic effect of CX-4945 alone or in combination with MMAE in Hodgkin lymphoma cell lines. HL cells lines were treated for 24 and 48 hours with CX-4945 and/or MMAE or the combination of both drugs. Apoptosis was detected through Annexin V propidium iodide (PI) assay by flow cytometry. Histograms shows alive non-apoptotic cells (Annexin V^neg^/PI^neg^). *Wilcoxon matched-pairs signed rank test* was used to compared paired data **(A)**. A qualitative WB (n=2), placed beneath each histogram, shows the cleavage of PARP protein expression in response to the heightened levels of apoptosis induced by the pharmacological treatments. In the middle panel **(B)** dose response curves of L-428, L-540, KM-H2, and HDLM-2 cell lines incubated for 72h with increasing concentrations of CX-4945 (green curves), and MMAE (orange curves) or their combination (dotted curves) are reported. Cell viability was assessed by trypan-blue exclusion assay. The curves were generated by maintaining a constant 1:1 ratio between the respective EC50 concentrations of CX-4945 and MMAE. In the lower panel **(C)**, EC50 values of CX-4945 and MMAE, used alone or in combination in L-428, L-540, KM-H2, and HDLM-2 cell lines incubated as in **(B)**. A combination index (CI) < 1 means a synergistic effect. Experiments were performed in triplicate. *p<0.05. *Wilcoxon matched-pairs signed rank test*.

To this aim, HL cell lines were treated with 5µM CX-4945 and 5nM MMAE, according to the literature (20), or a combination of both drugs for either 24 or 48 hours duration. As shown in **Figure 5A**, combination of CX-4945+MMAE significantly decreased the proportion of viable cells in comparison to MMAE. After 24 hours of *in-vitro* treatment, the percentage of viable cells, i.e. AV- PI-, decreased from 90.1%, 87.3%, 87.9%, and 94.8% with MMAE alone to 82.7% (*p*=0.12), 70.6% (*p*<0.05), 63.3% (*p*<0.05), and 85.9% (*p*<0.05) when treated with CX-4945 + MMAE in L428, L540, HDLM-2, and KM-H2, respectively (determined by the Wilcoxon matched-pairs signed rank test, **Figure 5A**). After 48 hours of treatment the rate of alive cells decreased from 79%, 62.2%, 66.5%, and 80.3% with MMAE alone to 64.3% (*p*=0.052), 51.8% (*p*=0.051), 48.4% (*p*<0.05), and 75.5% (*p*<0.05) with CX-4945+MMAE in L428, L540, HDLM-2, and KM-H2, respectively (*Wilcoxon matched-pairs signed rank test*, **Figure 5A**). The cell lines tested responded differently to the combination treatment of CX-4945 and MMAE. Specifically, KM-H2 and L-428 cells exhibited the lowest sensitivity, whereas HDLM-2 cells demonstrated the highest sensitivity to the combination treatment. The increased apoptotic effect resulting from the combination of CX-4945 and MMAE was further supported by WB analysis, revealing a decreased full-length PARP band and an increased cleaved protein band compared to cells treated exclusively with MMAE.

The possible synergism between CX-4945 and MMAE in reducing cell viability was evaluated using the Chou-Talalay method through the evaluation of trypan blue exclusion. The EC50 for each cell lines of the single treatment (CX-4945 or MMAE) and the EC50 for the drugs used in combination are reported in **Figure 5B**. The combination effect analyses identified that the combination index values were all below 1 for each cell line, indicating a synergistic cytotoxic effect between CX-4945 and MMAE. In particular, the obtained combination indexes were 0.91, 0.78, 0.67, and 0.23 for L-428, L-540, KM-H2, and HDLM-2, respectively (**Figure 5C**).

## Discussion

In this work, we characterized the role of protein kinase CK2 in cHL. In recent years, CK2 is emerging as an increasingly interesting target in hematological malignancies, being a key player in the regulation of proliferation, angiogenesis, secretion of growth factors, invasiveness and resistance to therapies (12). Previously, we and other groups demonstrated that both CK2α and CK2β were overexpressed and essential for neoplastic cell growth. Inhibition of CK2 triggered apoptosis in neoplastic cells of multiple myeloma (30), acute leukemias (26), and non-Hodgkin lymphomas (18, 25), without a significant impact on normal lymphocytes (31). However, the role of CK2 in cHL is unknown and has not been investigated yet.

In this study we demonstrated that CK2α was overexpressed in HL cell lines as compared to normal B-lymphocytes as assessed by WB and confocal microscopy. These data were also confirmed by IHC on primary samples of cHL patients, confirming that the catalytic α subunit was highly expressed, while the CK2β subunit was expressed at lower intensity. This aspect is remarkable, since, to our knowledge, cHL is the first hematological malignancy with an imbalance between α and β subunits. The expression of CK2α or β did not correlate with histological variants, age, stage, and outcome, suggesting that this unbalance expression of CK2α or β occurs in almost all cases and is likely to play a role in cHL development. Additionally, RT-qPCR analysis revealed a significant difference in mRNA expression levels only for the α subunits in the L-428 and L-540 cell lines when compared to healthy B cells. Therefore, we hypothesized that this unbalance could be the result of post-translational events in cHL. We investigated whether CK2β could be degraded by the proteasome. Consequently, we therefore immunoprecipitated all polyubiquitinated proteins following proteasome inhibition with bortezomib and assessed the presence of CK2β by immunoblotting. We observed an increase in the intensity of the CK2β subunit upon inhibiting the proteasome in all HL cell lines, indicating its degradation via the proteasome-dependent pathway. In line with our findings, recent studies have demonstrated that CK2 is active in the absence of the regulatory β subunit and that CK2β subunit influences substrate specificity, since there are proteins whose phosphorylation is specifically catalyzed by either the free α catalytic subunits, such as Akt S129, or CK2 holoenzyme through its N-terminal acid loop (32–34). In the latter case, CK2β acts as a docking platform for downstream substrates (35).

Of interest, our group has recently reported the first B-cell specific knockout mouse of CK2β, showing NOTCH2-mediated increase of marginal zone B cells and a decrease of follicular B cells (16). In addition, B cells lacking CK2β have an impaired signaling downstream to the B-cell receptor, toll-like receptor and CD40 (16). Since HRS are likely derived from crippled CD30+ germinal center B lymphocytes rescued by apoptosis, we can speculate that a skewed expression of CK2 subunits might be present also in CD30+ B cells or might be necessary for acquired the Hodgkin-phenotype as compared with other lymphomas where usually both subunits are overexpressed (25, 36).

Furthermore, pivotal signaling molecules in cHL, namely NF-kB, PI3K/AKT and STAT3, showed higher levels of their total protein compared to the control and were found to be phosphorylated at CK2 specific targets and therefore constitutively activated. Since AKT, NF-kB and STAT3 play a central role in HL, their basal phosphorylation suggests an important role of CK2 mediating a pro-survival function in HL (**Figures 2C–F**).

The CK2α inhibitor CX-4945/silmitasertib demonstrated a significant increase in *in vitro* apoptosis in the cHL cell lines tested, demonstrating a time- and dose-dependent response. Mechanistically, CX-4945 administration led to a decrease in the phosphorylation of the CK2 targets AKT, NF-kB and STAT3. Furthermore, PD-L1 receptor overexpression is known to be one of the main mechanisms by which HRS cells elude the immune response. PD-L1 gene is known to be amplified in most cases of cHL (37) and regulated by STAT3 and NF-κB transcription factors, both of which are activated by CK2 (11, 15). Our data demonstrate for the first time a novel CK2 mediated regulation of PD-L1 (CD274) in HL, since CK2 chemical inhibition with CX-4945 leads to the downregulation of PD-L1, possibly mediated through the impairment of STAT3 and NF-κB transcriptional activity. These findings suggest a potential indirect contribution of CK2 in inducing T-cell exhaustion and, consequently, contributing to an immunosuppressive microenvironment in HL.

The importance of targeting more pathways at the same time is highlighted by phase I-II clinical trials evaluating the clinical activity of JAK2 and/or PI3K inhibitors in relapsed refractory patients with HL (8, 38). A phase 1 dose-escalation/expansion study evaluated the safety and efficacy of dezapelisib, a new selective PI3Kδ inhibitor, as monotherapy or in combination with itacitinib, a selective JAK1 inhibitor, in adult patients with relapsed B-cell lymphomas. The combination of itacitinib and dezapelisib provided promising activity, resulting in an ORR of 67% in cHL compared to 29% in monotherapy (38). These clinical findings support the potential effectiveness of combining more pathway inhibitors as a relevant treatment strategy for highly pretreated HL patients. Accordingly, targeting CK2 would simultaneously switch off three key relevant survival signaling pathways. To this regard, we have also observed a synergistic effect between CX-4945/Silmitasertib and MMAE, a microtubule-disrupting agent conjugated with the anti-CD30 monoclonal antibody brentuximab vedotin. Although the CD30 overexpression is a common hallmark of HL and BV/MMAE has demonstrated clinical efficacy in the treatment of naive and relapsed patients, a small subset of triple refractory patients with cHL is emerging; therefore, it is of crucial importance to introduce potential pharmacological combinations to enhance therapy effectiveness. Silmitasertib (39) is a promising orally bioavailable selective inhibitor of protein kinase CK2. Despite CK2 significant impact on the human phosphoproteome, the inhibition of this kinase has been well tolerated in Phase I clinical trials (40). CX-4945 does not impinge on CD30 expression levels on the cell membrane as elucidated by the dose-response curves in each HL cell line tested in the present manuscript. Our data demonstrated that the mean EC50 value for individual treatments (CX-4945 or MMAE) undergoes a notable reduction upon their concomitant administration. This implies a prospective improvement in treatment tolerability, even at lower doses, all while upholding a precisely targeted cytotoxic effect designed to impair the tumor cell growth. Several *in vitro* studies like Martins et al. (41) in chronic lymphocytic leukemia, or Manni et al. (18) in mantle cell lymphoma, suggest that combination therapies with CX- potentially increase treatment response, particularly for drug-refractory patients. Furthermore, the inhibition of CK2 might contribute to achieve a synergistic treatment effect, even when used in combination with checkpoint inhibitors.

In this study, we have provided strong evidence that, in accordance with the paradigm known as “non-oncogene dependence” (18, 42), overexpression of CK2α is believed to be responsible for pivotal mechanisms of cell proliferation and survival also in cHL.

## Conclusions

We herein have demonstrated that cHL is likely the first hematological malignancy to exhibit an aberrant expression of CK2 subunits, with CK2α being overexpressed and CK2β downregulated in HRS cells, both in HL cell lines and primary lymph nodes from cHL patients. HRS cells exhibit a pronounced dependency on CK2α activity, since *in vitro* treatment with CX-4945/silmitasertib led to the dephosphorylation of AKT-S129 and S473, NF-κB-S529, and STAT3-S727, ultimately resulting in synthetic lethality in HL. Moreover, CX-4945 induced the downregulation of PD-L1/CD274, but not of CD30, and displayed a synergistic response with MMAE, which might have a relevant clinical implication. Further studies on CK2 protein will improve our understanding on HL pathogenesis.

## Data availability statement

The raw data supporting the conclusions of this article will be made available by the authors, without undue reservation.

## Ethics statement

The studies involving humans were approved by ethic committee of the Padova University Hospital (protocol # 4,089/AO/17). The studies were conducted in accordance with the local legislation and institutional requirements. The participants provided their written informed consent to participate in this study. Written informed consent was obtained from the individual(s) for the publication of any potentially identifiable images or data included in this article.

## Author contributions

ER: Data curation, Formal analysis, Investigation, Methodology, Validation, Writing – original draft. FF: Data curation, Formal analysis, Investigation, Methodology, Conceptualization, Project administration, Supervision, Writing – review & editing. NM: Formal analysis, Investigation, Methodology, Supervision, Writing – review & editing. MP: Formal analysis, Investigation, Methodology, Writing – review & editing, Data curation. FS: Data curation, Formal analysis, Investigation, Methodology, Writing – review & editing, Supervision. GC: Formal analysis, Writing – review & editing. VT: Data curation, Formal analysis, Investigation, Methodology, Supervision, Writing – review & editing, Conceptualization. LQT: Data curation, Formal analysis, Investigation, Methodology, Supervision, Writing – review & editing. AC: Data curation, Formal analysis, Investigation, Methodology, Writing – review & editing. CC: Data curation, Formal analysis, Investigation, Methodology, Writing – review & editing. VR: Data curation, Formal analysis, Investigation, Methodology, Writing – review & editing, Project administration. AS: Data curation, Formal analysis, Investigation, Methodology, Writing – review & editing. FA: Data curation, Formal analysis, Investigation, Methodology, Writing – review & editing. ND: Data curation, Formal analysis, Investigation, Methodology, Writing – review & editing. SM: Data curation, Formal analysis, Investigation, Methodology, Writing – review & editing, Supervision, Validation, Visualization. MF: Methodology, Supervision, Visualization, Writing – review & editing, Project administration. FP: Methodology, Project administration, Supervision, Visualization, Writing – review & editing, Investigation, Resources, Validation. LQ: Project administration, Resources, Supervision, Visualization, Writing – review & editing, Funding acquisition. AV: Writing – review & editing, Conceptualization, Data curation, Formal analysis, Funding acquisition, Investigation, Methodology, Project administration, Resources, Software, Supervision, Validation, Visualization, Writing – original draft.
