# Supplementary material for: Protein kinase CK2α is overexpressed in classical hodgkin lymphoma, regulates key signaling pathways, PD-L1 and may represent a new target for therapy
Source: Front Immunol. 2024 May 14;15:1393485. doi: 10.3389/fimmu.2024.1393485 (PMC11130512; doi:10.3389/fimmu.2024.1393485)

**SUPPLEMENTARY MATERIALS**

**HL cell lines features.**

Table 1 and Table 2 show respectively the immunophenotypic validation, performed in our laboratory, and the clinical features of L-428, L-540, HDLM-2 and KM-H2 HL cell line.

|  | **CD3** | **CD4** | **CD14** | **CD15** | **CD19** | **CD25** | **CD30** | **HLA-DR** |
| --- | --- | --- | --- | --- | --- | --- | --- | --- |
| **L-428** | **-** | **-** | **-** | **+** | **-** | **-** | **+** | **-** |
| **L-540** | **-** | **-** | **-** | **+** | **-** | **+** | **+** | **-** |
| **HDLM-2** | **-** | **+** | **-** | **+** | **-** | **+** | **+** | **+** |
| **KM-H2** | **-** | **-** | **+** | **+** | **-** | **-** | **+** | **+** |

**Table S1. Immune-phenotypic characterization of HL cell lines.**

|  | L-428 | L-540 | HDLM-2 | KM-H2 |
| --- | --- | --- | --- | --- |
| HL CELL SUBTYPE | Nodular sclerosis, stage IVB refractory terminal | Nodular sclerosis, stage IVB | Nodular sclerosis, stage IV | Mixed cellularity, stage IV at relapse |
| ORIGIN | Pleural effusion | Bone marrow | Pleural effusion | Pleural effusion |
| AGE | 37 y.o. | 20 y.o. | 74 y.o. | 37 y.o. |

**Table S2. Clinical characteristics of HL cell lines.**

**Assessment of drug concentration-effect and calculation of the combination index (CI).**

L-428, L-540, HDLM-2, and KM-H2 HL cell lines were plated into 48 well plates (2*10^5^ cells/ml) in appropriate culture medium. CX-4945, MMAE were added at different concentrations: CX-4945 ranging from 1μM to 25μM and of MMAE ranging from 0.05nM to 10nM for 72 h alone or in combination. Cell viability was measured through Trypan blue exclusion dye assay. The concentration of the single drug able to kill the 50% of cells (EC50) was determined by fitting the dose-response curve utilizing GraphPad Prism software. To calculate the Combination Index (CI), cells were treated with a combination of CX-4945 and MMAE using the method of constant ratio drug combination proposed and described by Chou and Talalay^1^.


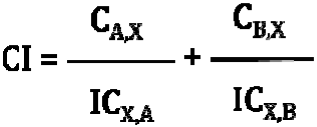
 The two drugs were used at a constant ratio of their concentrations. The concentrations used corresponded to 0.25, 0.5, 1, 2, 4 times the EC50 of each agent. The CI was calculated using the formula:

where CA,X and CB,X are the concentration of drug A and B used in combination to achieve x % drug effect. ICX,A and ICX,B are the concentrations for single agents to achieve the same effect. A CI of less then, equal to, or more than 1 indicates synergic, additive or antagonistic effect, respectively.

**SUPPLEMENTARY REULTS**

**Comparison of CK2 mRNA levels**

To study whether the unbalance between CK2 subunits were associated with skewed levels of CK2 mRNA, by RT-PCR we analyzed the mRNA of CK2 catalytic (α - CSNK2A1, and αI - CSNK2A2) and regulatory (β - CSNK2B) subunit (Figure S1-D).

The median mRNA levels of CSNK2A1/β-actin were as follows: 2.46±0.424 for L-428, 5.35±0.38 for L-540, 1.44±0.33 for HDLM-2, 2.02±0.69 for KM-H2, 1.3±0.168 for healthy donor B cells, and 4.1±0.76 for Kasumi-1, respectively. We observed a significant trend of higher mRNA levels for the α subunit only in the L-428 and L-540 cell lines when compared to normal B lymphocytes (*p*<0.01 and *p*<0.0001, respectively, unpaired *t* test). The median mRNA levels of CSNK2A2/β-actin were 1.48±0.96, 4.135±0.177, 0.98±0.54, 1.3± 0.40, 1.40±1.04, 6.00±1.56 for L-428, L-540, HDLM-2, KM-H2, healthy donor B cells, and Kasumi-1, respectively (*p* value: not significant, ns, *p*=0.138).

The median mRNA levels of CSNK2B/β-actin were 0.93±0.18, 2.43±1.6, 1.95±0.33, 1.76 ±0.42, 2.56 ±0.51, and 10.33±3.55 for L-428, L-540, HDLM-2, KM-H2, healthy donor B cells, and Kasumi-1 (*p*: ns, *p*=0.0577).

**Characteristics of the patients with HL**

The main clinical and histological characteristics of the 25 patients whose lympho nodes specimens were analyzed by tissue microarray, are summarized in Table S3. The median age at diagnosis was 35±16 years. 9 patients had an early stage (I- IIA) and 16 had an advanced stage disease (IIB-IV). 24 out of 25 patients were treated with ABVD-based treatment with or without involved field radiotherapy, the remaining with VEPEMB. Interim PET was positive, defined as DS>3, in 4 cases. After a median follow-up of 48 months 9 (36%) patients relapsed and 4 (16%) died.

**Table S3. Clinical and pathological features of HL cases.**

| **Patients** | **Gender** | **Age at diagnosis** | **Subtype** | **Stage** | **1L therapy** | **iPET** | **Relapse** | **ASCT** | **AlloSCT** | **Death** |
| --- | --- | --- | --- | --- | --- | --- | --- | --- | --- | --- |
| UPN-1 | 1 | 26 | NS | 2B | ABVDx6+RT | neg | yes | yes | no | no |
| UPN-2 | 0 | 19 | NS | 2A | ABVDx4+RT | neg | no | no | no | no |
| UPN-3 | 1 | 30 | MC | 4B | ABVDx6 | neg | yes | no | no | no |
| UPN-4 | 1 | 60 | MC | 4B | ABVDx6+RT | neg | yes | yes | no | yes |
| UPN-5 | 0 | 35 | MC | 4B | ABVDx6 | neg | yes | yes | no | no |
| UPN-6 | 1 | 69 | MC | 3B | AVDx6 | pos | yes | no | no | yes |
| UPN-7 | 0 | 46 | MC | 2B | ABVDx6 | neg | no | no | no | no |
| UPN-8 | 0 | 18 | NS | 2B | ABVDx6 | pos | yes | yes | no | no |
| UPN-9 | 0 | 28 | MC | 2A | ABVDx4+RT | neg | no | no | no | no |
| UPN-10 | 1 | 47 | MC | 1A | ABVDx2+RT | neg | yes | no | no | no |
| UPN-11 | 1 | 38 | NS | 4B | ABVDx6 | neg | yes | yes | yes | yes |
| UPN-12 | 0 | 28 | NS | 2A | ABVDx4+RT | neg | yes | yes | yes | no |
| UPN-13 | 1 | 18 | NS | 2B | ABVDx6+RT | neg | no | no | no | no |
| UPN-14 | 0 | 30 | NS | 2A | ABVDx4+RT | neg | no | no | no | no |
| UPN-15 | 1 | 30 | NS | 2B | ABVDx6+RT | neg | no | no | no | no |
| UPN-16 | 1 | 18 | NS | 2A | ABVDx4+RT | neg | no | no | no | no |
| UPN-17 | 1 | 42 | NS | 2A | ABVDx4+RT | neg | no | no | no | no |
| UPN-18 | 1 | 76 | NS | 3A | AVD | neg | no | no | no | no |
| UPN-19 | 1 | 26 | NS | 4B | ABVDx6 | neg | yes | no | no | no |
| UPN-20 | 1 | 21 | NS | 2A | ABVDx4+RT | neg | no | no | no | no |
| UPN-21 | 0 | 26 | NS | 2A | ABVDx4+RT | neg | no | no | no | no |
| UPN-22 | 1 | 20 | NS | 4A | ABVDx2+  4 BEACOPPesc | pos | yes | yes | no | no |
| UPN-23 | 1 | 30 | MC | 4A | ABVDx6 | neg | no | no | no | no |
| UPN-24 | 1 | 68 | LD | 4B | AVDx6 | neg | no | no | no | no |
| UPN-25 | 1 | 25 | MC | 4B | ABVDx2+  4 BEACOPPesc | pos | yes | no | no | no |

Gender reference: 0=female; 1=male, iPET: interim 18FDG positron emission tomography with a low-dose computer tomography scan; ASCT: autologous stem cell transplantation; AlloSCT: allogeneic stem cell transplantation. HL Subtype: SN: Nodular sclerosis; CM: Mixed cellularity; DL: Lymphocyte-depleted subtype. ABVD: doxorubicin/bleomycin/vinblastine-dacarbazine, RT: radio therapy; AVD: doxorubicin/vinblastine/dacarbazine; BEACOPPesc: bleomycin/etoposide/doxorubicin/cyclophosphamide/vincristine/procarbazine/ prednisone.

**Table S4. Densitometric values expressed and mean +/- standard deviation.**

| **cHL cell lines** | **p-AKT S473/AKT (A.U.)** | **AKT/ACT (A.U.)** | **p-AKT S473/ACT (A.U.)** |
| --- | --- | --- | --- |
| KMH2 | 0.71 ± 0.22 | 0.46 ± 0.06 (p<0.05) | 1.16 ± 0.04 (p<0.01) |
| L-428 | 0.67 ± 0.46 | 0.66 ± 0.13 (p<0.05) | 0.43 ± 0.10 |
| L-540 | 0.50 ± 0.05 | 0.68 ± 0.03 | 1.04 ± 0.1 (p<0.01) |
| HDLM-2 | 0.90 ± 0.23 | 0.58 ± 0.10 (p<0.05) | 0.91 ± 0.07 (p<0.01) |
| B cells (CONTROL) | 0.56 ± 0.27 | 0.31 ± 0.08 | 0.18 ± 0.03 |
|  |  |  |  |
| **cHL cell lines** | **p-AKT S129/AKT (A.U.)** | **AKT/ACT (A.U.)** | **p-AKT S129/ACT (A.U.)** |
| KMH2 | 0.69 ± 0.24 | 0.48 ± 0.05 (p<0.05) | 1.01 ± 0.27 |
| L-428 | 0.91 ± 0.25 | 0.71 ± 0.21 (p<0.05) | 0.62 ± 0.10 |
| L-540 | 0.67 ± 0.08 | 0.60 ± 0.04 | 0.97 ± 0.12 (p<0.05) |
| HDLM-2 | 0.59 ± 0.03 | 0.61 ± 0.11 (p<0.05) | 0.92 ± 0.01 (p<0.05) |
| B cells (CONTROL) | 1.09 ± 0.45 | 0.30 ± 0.07 | 0.34 ± 0.14 |
|  |  |  |  |
| **cHL cell lines** | **p-STAT3 S727/STAT3 (A.U.)** | **STAT3/ACT (A.U.)** | **p-STAT3 S727/ACT (A.U.)** |
| KMH2 | 1.54 ± 1.19 | 1.34 ± 0.19 (p<0.05) | 1.00 ± 0.1 (p<0.01) |
| L-428 | 0.66 ± 0.34 | 0.77 ± 0.12 (p<0.05) | 0.58 ± 0.14 (p<0.05) |
| L-540 | 0.88 ± 0.27 | 1.39 ± 0.44 (p<0.05) | 0.87 ± 0.13 (p<0.05) |
| HDLM-2 | 0.99 ± 0.08 | 1.02 ± 0.23 | 0.89 ± 0.18 (p<0.01) |
| B cells (CONTROL) | 1.02 ± 0.56 | 0.20 ± 0.00 | 0.27 ± 0.08 |
|  |  |  |  |
| **cHL cell lines** | **p-NF-kB (p65) S529/NF-kB (p65) (A.U.)** | **NF-kB (p65)/ACT (A.U.)** | **p-NF-kB (p65) S529/ACT (A.U.)** |
| KMH2 | 1.10 ± 0.45 | 1.69 ± 0.06 (p<0.05) | 1.43 ± 0.02 (p<0.01) |
| L-428 | 1.17 ± 0.04 | 0.68 ± 0.51 | 0.61 ± 0.27 |
| L-540 | 1.26 ± 0.18 (p<0.05) | 1.61 ± 0.34 | 2.12 ± 0.15 (p<0.01) |
| HDLM-2 | 1.29 ± 0.20 | 1.11 ± 0.06 (p<0.05) | 1.20 ± 0.16 (p<0.01) |
| B cells (CONTROL) | 0.81 ± 0.09 | 0.41 ± 0.1 | 0.34 ± 0.13 |

CHl = classical Hodgkin lymphoma; A.U. = arbitrar units.

**SUPPLEMENTARY REFERENCES**

1. Chou, T.-C. Theoretical Basis, Experimental Design, and Computerized Simulation of Synergism and Antagonism in Drug Combination Studies. *Pharmacol. Rev.* **58**, 621–681 (2006).

**LEGEND TO SUPPLEMENTARY FIGURES**

**Figure S1.** Picture of histological preparations of the tissue microarray (A), and immunohistochemistry of CK2 α (B) and β (C) subunits in patient-derived Hodgkin and Reed-Sternberg cells. Arrows indicate Hodgkin and Reed-Sternberg cells.

**Figure S2.** Progression-free survival (PFS) in the investigational low grade (0-1) *versus* high grade (2-3) of CK2β protein expression. Median PFS: 27 months; n.r., *p*=0.0421. Logrank test (A). Histograms depict the mean fluorescence intensity (MFI) of CD30 and CD20 expression in the four HL cell lines, with or without 10μM CX-4945 treatment for 24 and 48 hours (B). mRNA expression levels of CK2 subunit genes. Histograms depict mRNA levels of *CSNK2A1* (CK2α), *CSNK2A2* (CK2α^I^), and *CSNK2B* (CK2β) in HL cell lines, normal B lymphocytes (purified from n=3 Buffy-coat), and Kasumi-1 cell line. B cell: normal B lymphocytes. *p=*ns: not significant. ***p*<0.01, *****p*<0.0001, *Unpaired t test* (C).


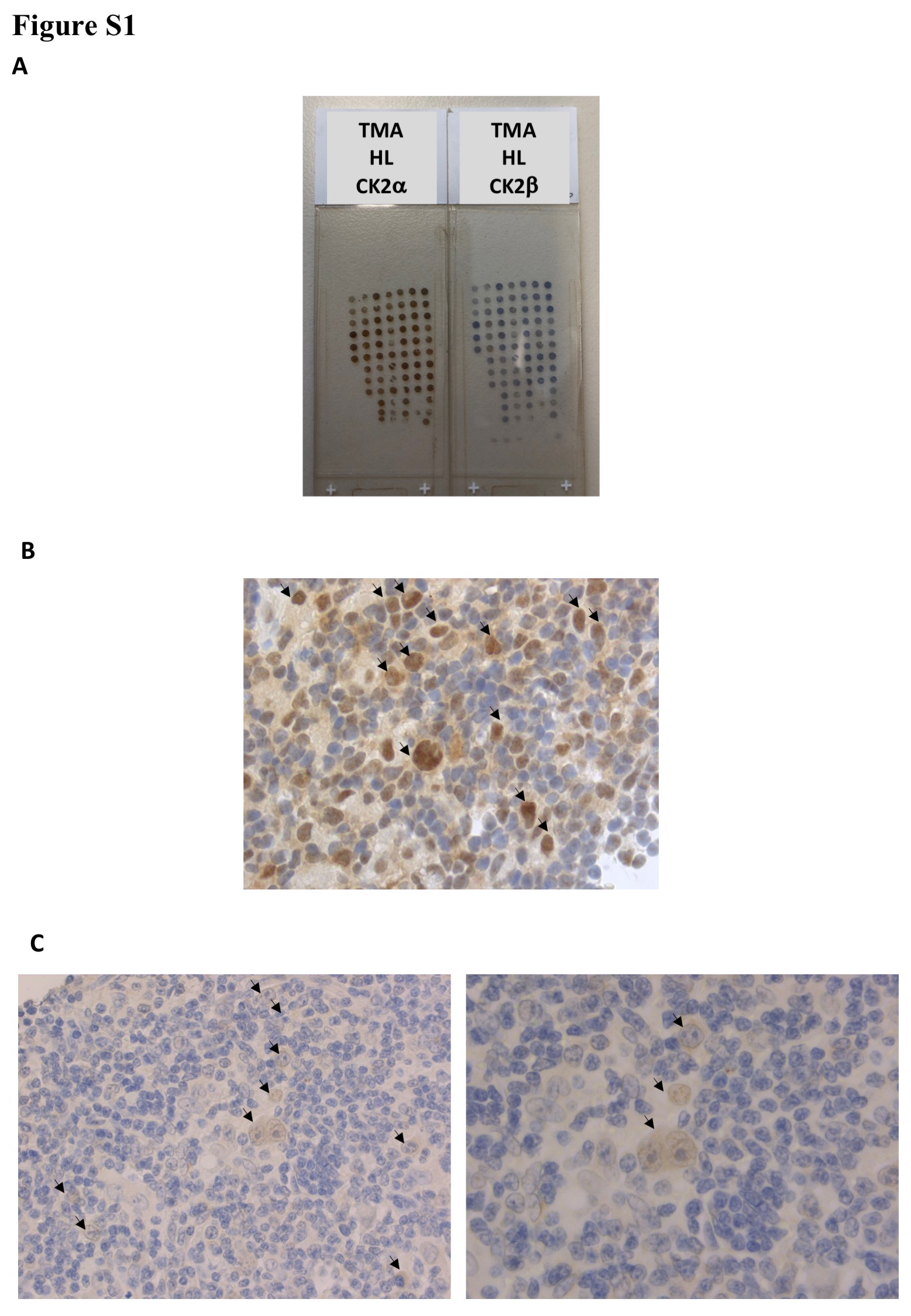


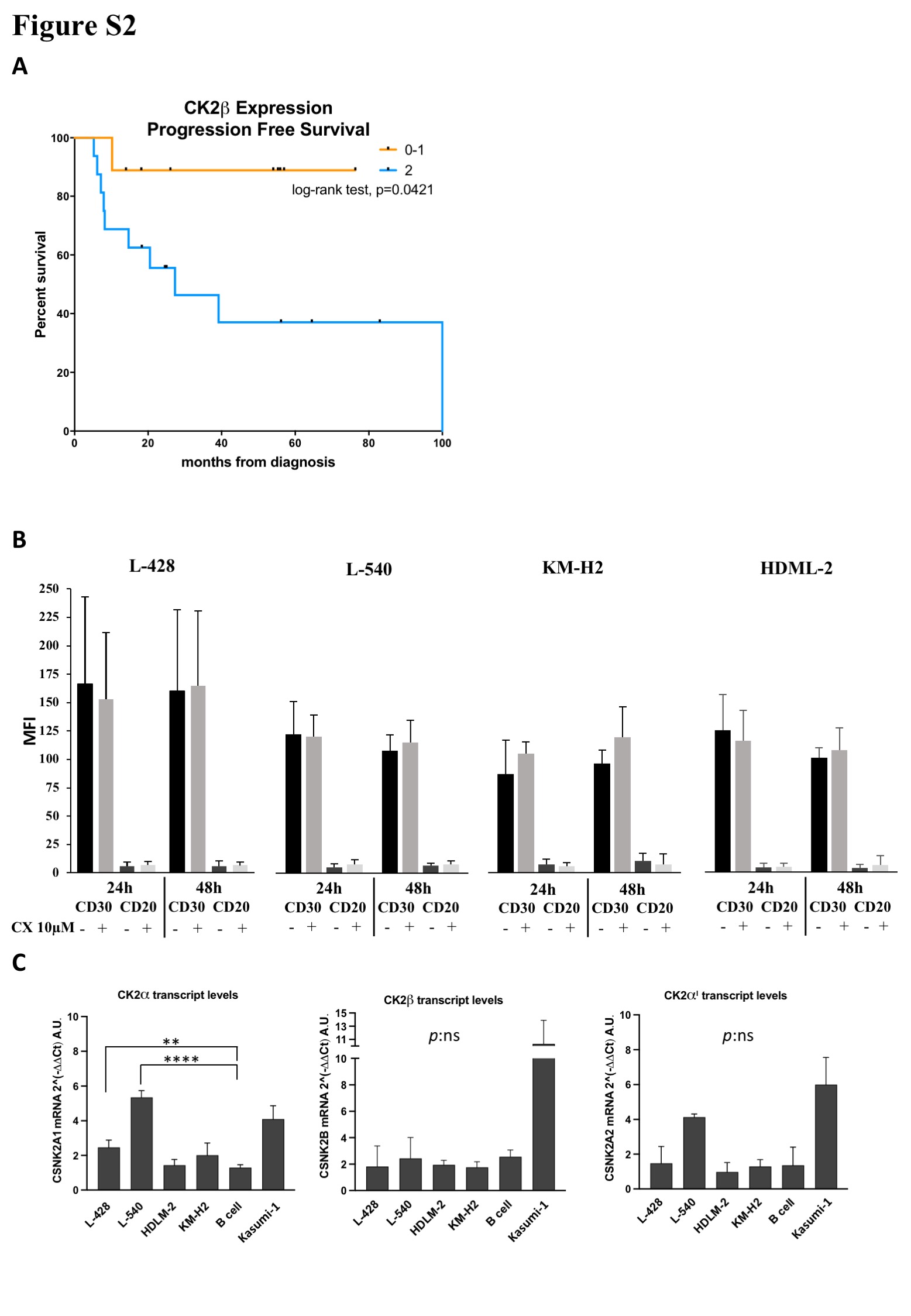

Supplement: Supplementary file 1 [file DataSheet_1.docx]
